# Physically inactive adults are the main users of sports dietary supplements in the capital of Brazil

**DOI:** 10.1007/s00394-022-02799-x

**Published:** 2022-01-31

**Authors:** Lara Pereira Saraiva Leão Borges, Alessandra Gaspar Sousa, Teresa Helena Macedo da Costa

**Affiliations:** Department of Nutrition, School of Health Science, Universidade de Brasília, Brasília, Federal District Brazil

**Keywords:** Food supplements, Prevalence, Exercise, Physical activity

## Abstract

**Purpose:**

This cross-sectional study aimed to provide estimates of dietary supplements (DS) use and to examine the relationship between sports dietary supplements (SDS) use and sociodemographic and lifestyle characteristic, body mass index and total daily physical activity (PA) in the adult population of Brasília, Brazil.

**Methods:**

DS use was collected from 506 adults via a Food Frequency Questionnaire. DS were divided into multivitamin-minerals, electrolyte drinks, energy, protein, partial meal replacements, creatine, caffeine, and others. Electrolyte drinks, energy, and protein supplements were considered as SDS. PA was estimated from a 24-h PA recall, and total MET (metabolic equivalents)/day and MET-h/day were calculated. Participants were categorized as physically inactive or active according to MET-h/day.

**Results:**

DS were used by 68% of adults; multivitamin-minerals (38%) and protein supplements (29%) were the most commonly used products. SDS use was associated with the highest socioeconomic level, younger age, and male gender, but not with PA. Also, most SDS users were physically inactive.

**Conclusion:**

These findings indicate that SDS may be used unnecessarily by adults in Brasília. Specific recommendations and control procedures for the use of SDS are warranted.

## Introduction

The definition and regulation of dietary supplements (DS) are not globally unified and differ from country to country [1]. In Brazil, the Brazilian Society for Food and Nutrition (SBAN) defines DS as products that are composed of concentrated nutrients or dietary substances such as vitamins, minerals, carbohydrates, proteins and amino acids, fatty acids, herbs and extracts, probiotics, enzymes, carotenoids, phytosterols or any other substance that has a nutritional, metabolic or physiological effect [2].

In the United States, DS are regulated by the Food and Drug Administration (FDA), which is responsible for monitoring dietary supplement products only after they enter the marketplace. DS manufacturers do not need FDA’s approval for selling these products [3], which contributes to the wide variety of DS products that are currently available on the market. In contrast, in Europe, a new product must be licensed by the national food safety authority [4]. In Brazil, the National Health Surveillance Agency (ANVISA) is the regulatory agency that oversees the registration of new foods/ingredients and products with functional or health claims before they are marketed [5]. Also, ANVISA regulates the use of sports dietary supplements (SDS) that include electrolyte drinks, energy, creatine, caffeine and protein supplements. Therefore, the list of ingredients or substances permitted or prohibited for use in DS may also vary from country to country.

People use dietary supplements to help meet their nutritional requirements when food consumption is insufficient, scarce, or inadequate. However, a considerable number of individuals from specific groups and populations in different countries take supplements. Studies with college students in Japan (*n* = 9,066) [6], a representative sample of Dutch population (*n* = 1544) [7], students in Croatia (*n* = 910) [8], fitness center users in Switzerland (*n* = 417) [9], the adult population (*n* = 11,956) in the United States [10], revealed that most individuals or participants used DS without a specific medical need or prescription issued by a licensed health practitioner.

The high prevalence of DS intake may be explained by the widespread belief that these products could improve consumer health [1, 10]. However, dietary supplements can also be harmful due to adverse effects and excessive intake of nutrients that can be toxic [1, 11]. Thus, because of their potentially beneficial and adverse effects and increasing use, interest in supplement use has increased in recent years. In the United States, for example, the prevalence of supplement usage increased by 25% over a 40-year period and over half of adults (52%) consumed some type of supplement in 2011‒2014 [12].

Studies in Germany, Australia, the Netherlands and Denmark have indicated that supplement users tend to be more physically active than nonusers [7, 13–15]. This finding is expected because DS are commonly used by individuals with increased nutritional demands from vigorous physical activity (PA) [16].

People living in Brasilia, the capital of Brazil, have sociodemographic characteristics that may influence DS use, such as higher education level, higher income, and higher prevalence of physically active individuals compared to other cities in Brazil [17, 18]. However, little is known about whether these sociodemographic and behavior characteristics are associated with DS use as observed in other countries. Thus, this study aimed to provide estimates of DS use and to examine the relationship between SDS use and sociodemographic and lifestyle characteristics, body mass index (BMI) and total daily PA in the adult population of Brasília, Federal District (FD), Brazil.

## Materials and methods

This study is part of a large project that investigated dietary intake and PA level in the adult population of Brasília. Detailed information about the project design and sample profile can be found elsewhere [19]. Briefly, sample size was calculated using an alpha error of 5% with a confidence level of 95% and assuming that 80% of the population engaged in < 150 min of PA per week, which is based on a Brazilian survey (Vigitel, 2012) that reported a prevalence of 12.6% (95% CI 10.5‒14.7) of individuals who undertook more than 150 min of PA per week. Sampling was performed using a cluster sampling design with households randomly selected from the 4 sanitary regions of central Brasília, yielding a sample size of 250 households and 500 individuals, assuming that each household had 2 adults. The final sample comprised 506 individuals, of whom 363 (72%) were recruited from a replacement sample of the same sample clusters. Replacements were required for vacant households and residents who refused to participate or did not meet the inclusion criteria. We interviewed residents aged ≥ 20 years of both genders who accepted our invitation to participate in the project. Pregnant women, nursing mothers, and individuals with disabilities or infirmities that prevented the administration of questionnaires or who could not have their anthropometric measurements taken were excluded.

All procedures were approved by the Ethics Committee of the University of Brasília School of Health Sciences (FS/UnB) (protocol number CAAE 48418315.4.0000.0030, advice n° 1.657.099) in accordance with the National Health Council (CNS) resolution (466/12) on research involving human beings. All participants provided written informed consent before participating in the study. Interviews were conducted by trained dieticians and graduate nutrition students.

Participants responded a sociodemographic questionnaire. For this study, we used the following variables: age in years, gender (male or female), socioeconomic class categorized according to monthly family income into classes A (R$ 23,345.11/US$ 6001.31), B (R$ 8011.35/US$ 2059.47), and C–E (R$ 1788.44/US$ 459.75) [20], and smoking (yes or no).

Body weight and height were measured in duplicate using a digital weight scale (WISO, São José, SC, Brazil) and Personal Caprice vertical stadiometer (Sanny, São Paulo, SP, Brazil). For these assessments, the participants were placed in a standing position, with relaxed arms, head in the horizontal plane and wearing light clothes. Average weight and height were obtained to calculate the body mass index (BMI), and the World Health Organization (WHO) cut-offs were used to classify the respondents [21].

DS use in the previous year prior to the household interview was collected via a Food Frequency Questionnaire (FFQ) with a specific session for supplements, which is a self-reported questionnaire. Participants were asked about the frequency of DS use (daily, weekly, monthly, or annual), type of supplement, manufacturer, and dose consumed. A photograph of the supplement container was taken for each DS reported by participants. Dietary supplements were divided into eight groups according to the ANVISA classification [16, 22] as follows: multivitamin-minerals (MVMs), electrolyte drinks, energy, protein, partial meal replacements (PMR), creatine, caffeine, and others (fatty acids and any other products that did not fit into the previous categories). Examples of supplements from each group were given by interviewers to help participants better recall the use and frequency of intake of the products they had taken.

Daily PA was estimated from a 24-h physical activity recall (24hPAR), which has been validated for the Brazilian population [23]. The 24hPAR was performed on two nonconsecutive days. Participants were asked to report all activities performed on the previous day, along with their intensity, on a one-hour time scale. The reported activities (including sleep, sedentary, occupational, travel, home, and leisure activities) with a minimum duration of 10 consecutive minutes were recorded and converted to metabolic equivalents of task (MET) according to the Compendium of Physical Activities [24].

Total MET/day was estimated from the sum of the mean of MET values from each hour of the day. For individuals with two 24hPAR, the average for total MET/day was calculated. Total MET/day was divided by 24 to calculate MET-h/day, which was used to categorize PA level of participants into sedentary (≥ 1.0 and < 1.4 MET-h/day), low active (≥ 1.4 and < 1.6 MET-h/day), active (≥ 1.6 and < 1.9 MET-h/day), and very active (≥ 1.9 MET-h/day) according to the Institute of Medicine (IOM) [25]. The measurement error associated with 24hPAR was addressed by applying a calibration equation to the total MET/day value of each day to compute corrected MET/day values. The calibration equation was developed with a subsample of this study and a complete description is available in Borges et al. [26].

Categorical variables are reported as absolute and relative numbers and numerical variables as mean, median, standard deviation (SD), and interquartile range (IQR) for physically inactive (MET-h/day < 1.6) and active (MET-h/day > 1.6) individuals. The Chi-square or Fisher’ exact test, when applicable, was used to compare the proportion of physically active and inactive individuals by sociodemographic characteristic. The Mann–Whitney test was used to compare total MET/day between physical activity level (PAL) groups. The Kruskal–Wallis test was used to examine demographic, socioeconomic, and lifestyle differences related to SDS use. For inferential analyses (regressions and the difference in the amounts of DS consumed between PAL groups), we followed the ANVISA classification of supplements for athletes, and those products with the highest total intake contribution (electrolyte drinks, protein and energy supplements) were referred to as SDS. Mean and median values for energy supplements were calculated separately for liquids and powders because they have different units of measure. The association of SDS intake (yes/no) with gender, socioeconomic class (A–E), smoking (yes/no), age (years), BMI (kg/m^2^), and daily PA (total corrected MET/day) was tested using a Poisson regression model. Simple linear regression analysis was used to determine the correlation between the amounts of SDS consumed in the past year and PA (total corrected MET/day) for each supplement subgroup. A 95% confidence interval (CI) is provided when applicable and a *p* value < 0.05 was considered statistically significant.

## Results

**Fig. 1:**
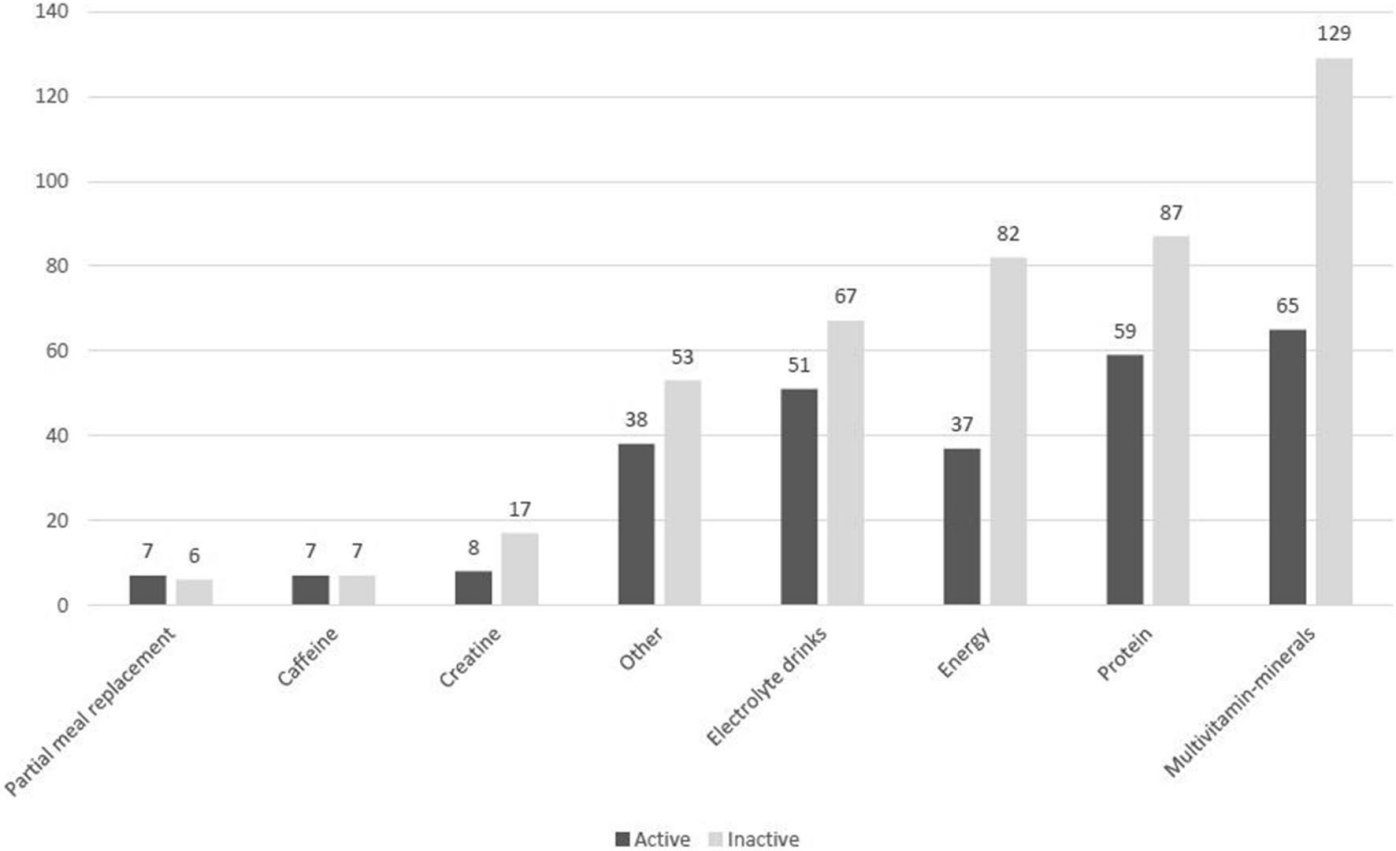
Absolute distribution of dietary supplement use for physically active and inactive adults in Brasília, Brazil, 2016‒2017

Over two-thirds of adults (68%, *n* = 345) reported using a DS product within the past year. Thirty-five percent of participants (*n* = 119) used one type of supplement, 28% (*n* = 97) two types, 20% (*n* = 70) three types, and 17% (*n* = 59) took four or more different types of DS. The most consumed type of supplement was multivitamins-minerals (38%, *n* = 194), followed by proteins (29%, *n* = 146), energy supplements (24%, *n* = 119), and electrolyte drinks (23%, *n* = 118). Figure 1 shows the distribution of the different types of supplements used by physically active and inactive individuals. The prevalence of individuals who used any type of DS, except PMR and caffeine, was higher for the physically inactive group.

The sociodemographic characteristics and prevalence of DS use per type of supplement for physically active and inactive individuals are summarized in Table 1. Sixty-three percent of DS users were physically inactive (217 individuals). Both PAL groups had similar characteristics: most individuals were women and had high socioeconomic status (class B) and educational attainment (university diploma). In contrast, the frequency distributions for age (*p* = 0.006), smoking status (*p* = 0.036), and BMI (*p* = 0.005) were significantly different in the two PAL groups. The prevalence of individuals 36‒52 years of age was significantly higher in the physically active group. Individuals classified as physically active were less likely to smoke. For BMI status, the prevalence of individuals with normal weight (BMI < 25 kg/m^2^) was significantly higher in the physically inactive group, whereas the prevalence of overweight (BMI 25‒29.9 kg/m^2^) was higher among physically active individuals. In addition, the prevalence of SDS use was higher among physically inactive individuals for the four types of sports supplements, but the difference was statistically significant only for liquid energy supplements.

**Table 1:** Sociodemographic and lifestyle characteristics and estimated prevalence (%) of sports dietary supplements use by physical activity level among adults in Brasília, Brazil, 2016‒2017 (*n* = 345)

Variable	Physical activity level				p*
	Inactive (*n* = 217)		Active (*n* = 128)		
	*N*	%	*N*	%	
Gender					
Female	129	59.4	69	53.9	0.315
Male	88	44.6	59	46.1	
Socioeconomic class					
A	50	23.1	32	25.0	0.762
B	114	52.5	69	53.9	
C–E	53	24.4	27	21.1	
Education level					
Elementary/high school	30	13.8	15	11.7	0.645
Undergraduate	101	46.6	65	50.8	
Graduate	86	39.6	48	37.5	
Age quartile (years)					
1st (20‒27)	74	34.1	31	24.2	0.006
2nd (28‒35)	46	21.2	35	27.3	
3rd (36‒52)	40	18.4	40	31.3	
4th (53‒87)	57	26.3	22	17.2	
Smoking					
No	190	87.6	121	94.5	0.036
Yes	27	12.4	7	5.5	
BMI (kg/m^2^)					
< 25	133	61.3	58	45.3	0.005
25‒29.9	56	25.8	55	43.0	
> 30	28	12.9	15	11.7	
Sports DS users					
Electrolyte drinks	67	56.8	51	43.2	0.090
Liquid energy supplements	65	71.4	26	28.6	0.050
Powder energy supplements	30	62.5	18	37.5	0.951
Protein supplements	87	59.6	59	40.4	0.276

Physical activity	Inactive (*n* = 217)		Active (*n* = 128)		
	Median	IQR	Median	IQR	p**
Total MET/day	33.1	29.2‒35.9	42.9	40.9‒47.5	< 0.001

Average household income by socioeconomic class: A = R$ 23,345.11/US$ 6001.31; B = R$ 8011.35/US$ 2059.47; C–E = R$ 1788.44/US$ 459.75

*BMI* body mass index, *DS* dietary supplement, *MET* metabolic equivalents of task, *IQR* interquartile range

*Chi-square/Fisher’s exact test

**Mann–Whitney test

Table 2 shows the estimated daily intake of SDS in the past year according to sociodemographic characteristics, BMI status, and PA level. Consumption of electrolyte drinks and protein supplements was significantly higher among men than women. In addition, the amount of protein supplement use was significantly higher among participants aged 20‒35 years than 53‒87 years, whereas consumption of liquid energy supplements was significantly higher among participants aged 28‒52 years and smokers. In contrast, no significant differences were found in the amount of powder energy supplement use for the variables analyzed. In addition, SDS intake was not significantly affected by education level, socioeconomic class, BMI status, and PA level.

**Table 2:** Amounts of sports dietary supplements intake in the previous year by sociodemographic and lifestyle characteristic, BMI, and physical activity level among adults in Brasília, Brazil, 2016‒2017

Variable	Protein supplements (g/day)		Electrolyte drinks (ml/day)		Powder energy supplements (g/day)		Liquid energy supplements (ml/day)	
	Median (IQR)	p*	Median (IQR)	p*	Median (IQR)	p*	Median (IQR)	p*
Gender								
Female	10.0 (2.9‒21.3)	0.006	7.5 (2.5‒21.46)	0.002	2.33 (1.45‒9.48)	0.427	8.4 (2.0‒32.6)	0.597
Male	21.3 (5.015‒36.43)		35.0 (11.0‒70.0)		3.65 (2.06‒12.5)		8.63 (4.73‒35.0)	
Age quartile (years)								
1st (20‒27)	12.9^a^ (7.2‒30.0)	0.042	35.0 (7.0‒70.0)	0.283	2.15 (2.06‒12.5)	0.162	7.5^a^ (2.5‒22.5)	0.031
2nd (28‒35)	19.35^b^ (3.0‒36.85)		15.0 (4.0‒35.0)		1.96 (0.34‒3.1)		17.5^a,b^ (4.75‒65.0)	
3rd (36‒52)	10.85 (2.93‒29.47)		15.0 (4.5‒28.23)		4.6 (1.85‒8.83)		8.7^c^ (7.5‒35.0)	
4th (53‒87)	10.0^a,b^ (0.65‒12.9)		15.0 (4.0‒35.0)		9.2 (3.5‒48.55)		1.38^b,c^ (0.5‒8.5)	
Education level								
Elementary/high school	15.0 (7.5‒30.0)	0.591	15.0 (7.0‒35.0)	0.796	2.1 (1.73‒11.8)	0.425	7.5 (3.0‒28.75)	0.647
Undergraduate	12.9 (2.94‒30.0)		15.0 (4.0‒70.0)		5.0 (2.15‒10.0)		8.0 (2.5‒32.5)	
Graduate	10.4 (2.6‒30.0)		15.0 (5.0‒35.0)		3.1 (1.42‒8.7)		13.75 (4.73‒35.0)	
Socioeconomic class								
A	11.53 (5.0‒30.0)	0.58	9.25 (2.5‒60.0)	0.286	5.0 (1.45‒17.1)	0.064	7.25 (2.5‒38.75)	0.675
B	12.0 (2.94‒30.0)		15.0 (5.0‒70.0)		4.6 (2.13‒10.0)		8.33 (4.73‒27.5)	
C–E	19.58 (4.2‒30.0)		15.0 (5.0‒35.0)		2.0 (0.4‒2.5)		17.5 (1.25‒35.0)	
Smoking								
No	12.9 (3.0‒29.47)	0.326	15.0 (4.0‒35.0)	0.089	3.65 (1.7‒10.0)	0.901	7.5 (2.0‒32.5)	0.037
Yes	28.5 (5.0‒39.48)		15.0 (15.0‒70.0)		2.6 (2.03‒10.0)		17.5 (7.5‒42.5)	
BMI								
< 25 kg/m^2^	12.9 (2.49‒30.0)	0.341	15.0 (4.0‒35.0)	0.068	2.33 (2.0‒8.4)	0.484	8.33 (2.0‒20.0)	0.495
25‒29.9 kg/m^2^	18.23 (6.24‒35.36)		15.0 (7.0‒70.0)		5.0 (1.42‒15.0)		12.5 (7.0‒35.0)	
> 30 kg/m^2^	10.0 (7.2‒17.4)		35.0 (15.0‒50.0)		10.0 (2.1‒10.0)		11.7 (4.63‒86.25)	
Physical activity								
Inactive	12.9 (4.2‒27.0)	0.613	15.0 (4.0‒35.0)	0.203	2.15 (1.7‒8.96)	0.096	9.0 (4.0‒35.0)	0.937
Active	12.9 (2.6‒36.85)		15.0 (5.0‒70.0)		8.4 (2.5‒15.0)		7.95 (2.5‒35.0)	

Average household income by socioeconomic class: A = R$ 23,345.11/US$ 6001.31; B = R$ 8011.35/US$ 2059.47; C–E = R$ 1,788.44/US$ 459.75

*BMI* body mass index, *IQR* interquartile range

*Kruskal–Wallis test

^a,b,c^Median values followed by the same letters are significantly different by the post-hoc test

Table 3 shows results for the Poisson regression model on the association of SDS use in the past year (yes/no) with gender, socioeconomic class, smoking, age, BMI, and daily PA (total corrected MET/day values). A positive and significant association was observed for socioeconomic class, whereas female gender and age were negatively associated with DS use. These results indicate that high-income younger men are the highest consumers of DS among adults in Brasília. In addition, daily PA was borderline significant for SDS use.

**Table 3:** Poisson regression model for the association of sports dietary supplement use in the previous year with demographic, socioeconomic and lifestyle characteristics, BMI, and daily physical activity among adults in Brasília, Brazil, 2016‒2017

	PR	95% CI	*p*
Intercept	0.91	0.491‒1.69	0.766
Gender			
Female	0.773	0.655‒0.914	0.002
Socioeconomic class			
A	1.344	1.066‒1.696	0.013
B	1.144	0.927‒1.411	0.623
Smoking			
No	1.173	0.919‒1.497	0.2
Age (years)	0.982	0.975‒0.988	< 0.001
BMI (kg/m^2^)	0.989	0.967‒1.012	0.34
PA (total MET/day)	1.007	1.0‒1.015	0.055

Average household income by socioeconomic class: A = R$ 23,345.11/US$ 6001.31; B = R$ 8011.35/US$ 2059.47. Male gender, socioeconomic class C, and yes for smoking are the reference parameters. BMI and PA are continuous variables

*BMI* body mass index, *PA* physical activity, *MET* metabolic equivalents of task, *PR* prevalence ratio, *CI* confidence interval

**Fig. 2:**
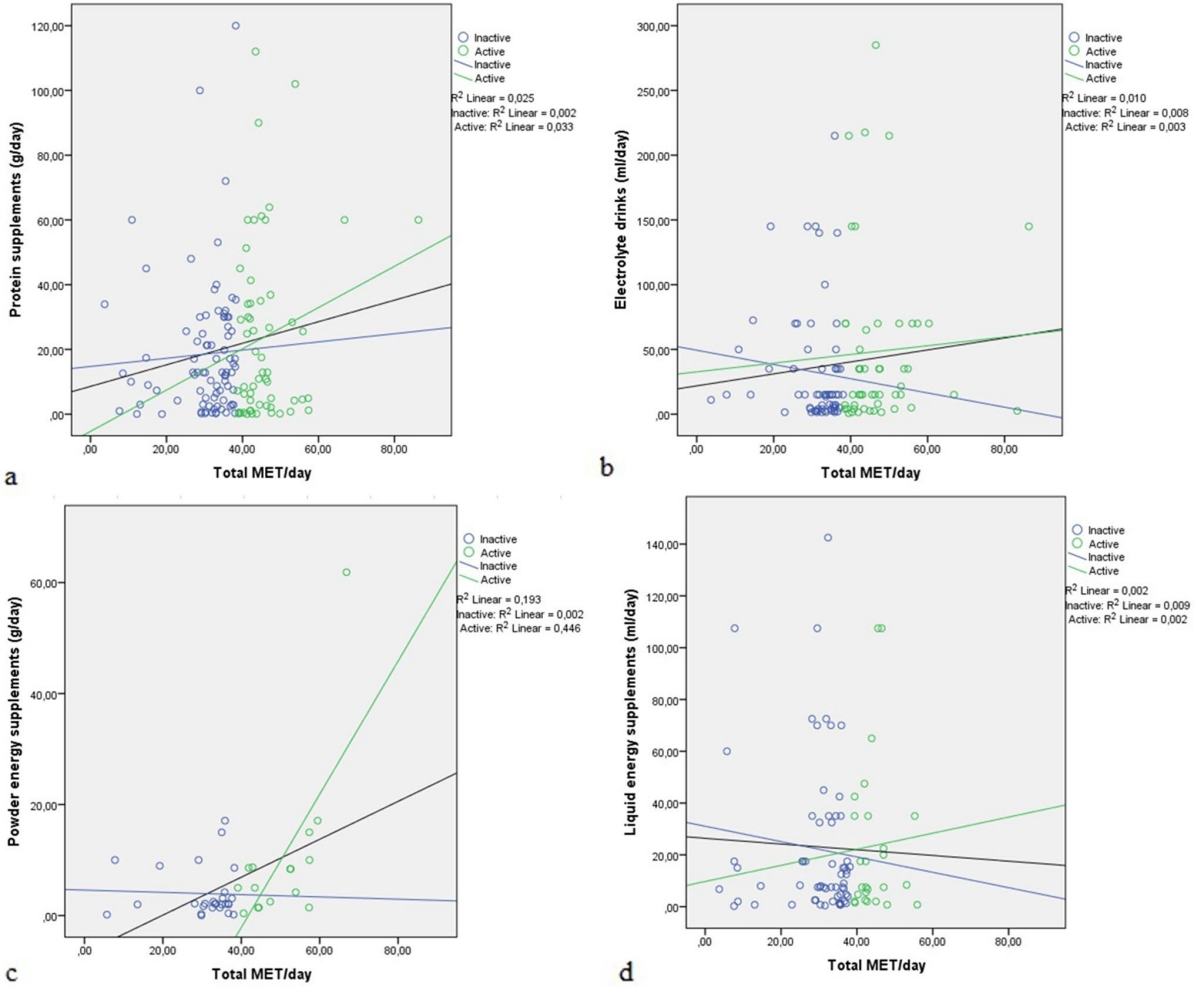
Scatter plots for the simple linear regression of the amounts of protein supplements (**a**), electrolyte drink supplements (**b**), powder energy supplements (**c**), and liquid energy supplements (d) consumed in the previous year (without outliers) on total MET/day among adults in Brasília, Brazil, 2016‒2017. Blue dots and lines: physically inactive individuals; green dots and lines: physically active individuals; and black lines: relationship for all consumers of the specified supplement. MET: metabolic equivalents of task

For the four types of SDS examined, there were outliers with very high intakes. Thus, we performed simple linear regressions between SDS intake and daily PA (total MET/day) with and without the outliers. No significant correlation was found with the entire data set and there was only a marginally significant correlation (*r* = 0.161, *p* = 0.052) for protein supplements. Similarly, when outliers were removed from the analyses, the regressions remained nonsignificant (*r* = 0.157, *p* = 0.06**)**. We also performed simple linear regressions between SDS use and PA for physically inactive and active individuals. However, no significant correlation was observed. The correlation coefficients of the linear regressions for all SDS users and physically inactive and active individuals are shown in Table 4. Scatter plots of the four linear regressions between amounts of each type of SDS consumed in the last year and total MET/day for all SDS users (black line), and physically active (green line) and inactive (blue line) individuals are shown in Fig. 2.

**Table 4:** Simple linear regressions of sports dietary supplement intake on physical activity for total, physically inactive, and physically active adults in Brasília, Brazil, 2016‒2017

	Total			Inactive			Active		
	Coefficient	SE	*p*	Coefficient	SE	*p*	Coefficient	SE	*p*
Protein supplements (g/day)									
Intercept	8.456	6.706	0.209	15.201	8.612	0.081	− 5.3	21.557	0.807
Total MET/day	0.332	0.175	0.06	0.104	0.275	0.706	0.637	0.462	0.173
Electrolyte drinks (ml/day)									
Intercept	21.869	17.32	0.209	49.379	24.577	0.049	32.674	46.395	0.485
Total MET/day (corrected)	0.462	0.433	0.288	− 0.55	0.776	0.481	0.336	0.953	0.726
Powder energy supplements (g/day)									
Intercept	− 3.814	6.386	0.553	6.952	7.786	0.379	− 25.055	22.956	0.289
Total MET/day (corrected)	0.312	0.163	0.062	− 0.046	0.244	0.852	0.762	0.47	0.123
Liquid energy supplements (ml/day)									
Intercept	26.404	10.611	0.015	31.031	10.883	0.006	9.735	59.089	0.871
Total MET/day (corrected)	− 0.11	0.299	0.713	− 0.362	0.352	0.308	0.311	1.324	0.816

*SE* standard error, *MET* metabolic equivalents of task

## Discussion

Results from this study indicate that over two-thirds of our sample take one or more DS, particularly MVMs and protein supplements, and most individuals who use SDS are physically inactive. This result points to the unnecessary use of electrolyte drinks, protein and energy supplements by consumers in Brasília.

The global prevalence of DS use is unknown due to the diversity of methodologies used and lack of available data in most countries [27]. Indeed, no national survey on DS use has been conducted in Brazil. Information on DS use is only available from a representative sample from the city of São Paulo, where the prevalence of DS use among adolescents, adults, and the elderly was estimated to be approximately 6% [28]. In a review by Rautiainen et al. [27], the prevalence of DS use varied from 22 to 53% in other countries, including the United States, Canada, South Korea, England, Sweden, Germany, and France. The representative sample of Dutch population presented similar prevalence to ours (62%) [7]. China has the lowest prevalence of DS use (0.71%), including the population aged 6 years or older [29], of all countries where DS use has been assessed by population surveys.

The prevalence of DS use in the adult population of Brasília was considerably higher than in previous studies, as over two-thirds of participants (68%) reported taking a supplement in the current study. The time of data collection and the duration of DS use analyzed could explain the differences in DS use between studies. The review by Rautiainen et al. [27] included studies that were conducted from 1993 to 2009 and used questionnaires that collected DS use within the previous 7 days (England), 2 weeks (Sweden), 4 weeks (Germany), 30 days (Canada and the United States), and 12 months (South Korea and France). The survey performed in São Paulo was conducted in 2007‒2008 and asked if participants consumed any type of supplement. If the answer was yes, the participant was asked about the frequency of use, either regular or sporadic, but not the duration of use [28]. Gong et al. [29] used data from the 2010‒2012 China Nutrition and Health Surveillance (CNHS) to estimate the national prevalence of DS use in the previous 30 days. In contrast, the current study was conducted in 2016‒2017 and assessed DS use in the previous year. The survey conducted in the Netherlands in 2013, considering also DS use in the last year, is the one that presented results closest to ours [7].

Multivitamin supplements alone or in association with minerals are the most frequently consumed supplement in many population groups [12–14, 28, 30–32]. A recent study of our research group that collected information on the dietary habits and nutritional status of the residents of Brasília using 24-h food recalls showed that 55% of DS users consumed some type of vitamin and mineral supplements, which contribute to achieving nutrient adequacy [33]. Moreover, protein supplements were the second most commonly consumed DS in the current study. A similar preference for protein supplements has been found in studies with professional and recreational athletes from different countries such as Saudi Arabia [34], Brazil [35], and Portugal [36]. A possible explanation for the high intake of protein supplements in our study is that 93% of users (136 out of 146) reported engaging in regular physical exercises. A study with Australian university students showed that the most frequent reported reason for consuming protein supplements is to enhance physical performance [37].

We identified gender, socioeconomic class and age as associated factors of SDS use. Participants that have the highest income (class A) presented 34% more chance to use SDS. This result is already expected, since individuals in class A have great purchasing power, facilitating acquisition. Similar findings are observed in studies performed in United States, China, France, Australia, and United Arab Emirates [12, 29–32]. On the other hand, women and older individuals presented less probability to consume SDS then their counterparts, which is in accordance with the profile of sports nutrition product users in the Netherlands [7].

This study also provides valuable information on the amount of SDS consumed in relation to gender, age, education level, socioeconomic status, smoking, BMI, and PA level. Results from the analysis showed that amount of protein and electrolyte supplements consumed was higher among men than women. In addition, older adults had a lower amount of protein and liquid energy supplements, whereas smokers reported a higher intake of liquid energy supplements. Previous studies showed that the use of dietary supplements is associated with female gender, older age, higher educational attainment, normal weight, and abstinence from smoking, but none of these studies assessed the amounts of DS consumed [10, 13, 14, 28, 30, 31, 38].

An alarming finding of our study was that most SDS users were classified as physically inactive (*n* = 217; 63% of 345) and the amounts of SDS consumed were not significantly different between physically active and inactive participants. The prevalence found in our study was substantially higher than sports nutrition products use in a Dutch population, still reporting a substantial 15% of participants using sport nutrition drinks [7]. Moreover, no association was found between PA and SDS use or the amount consumed in the past year. As seen in other studies [15, 37], the expected association between these variables is a positive one, whereby individuals with higher PA level are more likely to use SDS and have a higher use of these products. Our study provides additional information on the relationship between the amount of SDS used and PA (total MET/day). Even though the correlations did not reach statistical significance, electrolyte drinks and energy supplements showed an inverse relationship with PA for physically inactive users (Fig. 2), indicating that those with lower reported PA consumed higher amounts of these products. This result suggests that most participants in this study consume SDS unnecessarily, since these supplements are recommended for individuals that engage in vigorous exercise and need to support their higher energy and nutrient requirements [16].

The unnecessary use of energy, electrolyte drinks, and protein supplements may lead to excessive intake of energy, refined carbohydrates, and protein. Energy intake above normal requirements leads to body weight gain, which may progress to overweight or obesity, increasing the risk of developing noncommunicable diseases and the risk of mortality [25]. The excessive intake of refined carbohydrates has been associated with higher levels of blood triglycerides and LDL cholesterol, and higher BMI, particularly in individuals with metabolic disorders [25]. Moreover, there is evidence that, for healthy adults, diets higher in glycemic index and glycemic load are associated with a higher incidence of type 2 diabetes [39]. Consumption of protein at up to 2 g per kg body weight per day is usually well tolerated by healthy adults. However, excessive protein intake, especially from DS consumption, may lead to gastrointestinal distress and kidney function disorders [40] or the excess protein may be stored as body fat if a positive energy balance diet is maintained without a concurrent increase in physical workload.

It is important to highlight that few studies described the prevalence of DS use at the population level around the world. Few countries conduct national surveys that investigate the prevalence and factors associated with DS use and that monitor trends in supplement consumption over time [7, 10, 15, 29]. Moreover, most studies on DS use have been conducted with specific groups, such as gym users or athletes, that regularly use these products [6, 8, 9, 34–37].

The current study has some limitations. The cross-sectional nature of the data makes it impossible to infer causality between DS use and PA level. While our sample size and design were careful to cluster the four sanitary regions of Brasília, we cannot completely rule out the potential for self-selection bias, which limits the extrapolation of the results to the general population. DS data were obtained through a self-reported tool, which may produce results with under/overestimation, since it depends on respondent’s memory. Nevertheless, this study provides original information on long-term patterns of DS use in relationship to daily PA in a high-income population with high educational attainment.

## Conclusions

This study provides original information on DS use, type, and number of supplements taken by adults living in Brasília, the capital of Brazil. DS are used by over two-thirds of adults (68%); MVMs are the most frequently consumed supplement, followed by protein supplements. Results also indicate that the adult population consumes protein, electrolyte, and energy supplements unnecessarily. We believe that our findings can contribute to raising public awareness about the real need for consuming DS and the importance of consulting a qualified professional before starting using these products. Further studies are needed to describe the prevalence and factors associated with DS use, the reasons and needs for taking these products, and to monitor DS use over time, particularly at the population level.

## Data Availability

The authors make sure that all data and materials support published claims and comply with field standards.
